# Six Sessions of Sprint-Interval Training Did Not Improve Endurance and Neuromuscular Performance in Untrained Men

**DOI:** 10.3389/fphys.2019.01578

**Published:** 2020-01-28

**Authors:** Raphael Bertschinger, Louis-Solal Giboin, Markus Gruber

**Affiliations:** Human Performance Research Centre, Department of Sport Science, University of Konstanz, Konstanz, Germany

**Keywords:** sprint-interval training, neuromuscular function, voluntary activation, transcranial magnetic stimulation, peripheral nerve stimulation, maximal voluntary contraction

## Abstract

Previous research demonstrated that six sessions of cycling sprint-interval training (SIT) within a duration of only 2 weeks can increase endurance performance considerably. Primarily muscular mechanisms have been under investigation explaining such performance improvements. However, it has been shown in other exercise tasks that training-induced changes also occur at the level of the central nervous system. Therefore, we hypothesized to observe an enhanced neuromuscular performance in conjunction with an increase in endurance performance after 2 weeks of SIT. Therefore, we randomly assigned 19 healthy men (26 ± 5 years) to a control (*n* = 10) or a training group (*n* = 9), the latter performing a replication of the SIT protocol from Burgomaster et al. Before and after the training intervention, both groups performed a cycling endurance test until exhaustion. Neuromuscular function of the right vastus lateralis muscle was assessed before and after each endurance task by the means of maximal voluntary isometric contractions (MVCs). The variables of interest being MVC, voluntary activation was measured by peripheral nerve stimulations (VA_PNS_), by transcranial magnetic stimulation (VA_TMS_), as well as potentiated resting twitches (Q_tw,pot_). We did not find any significant differences between the groups in the control variable time to exhaustion in the endurance task. In addition, we did not observe any time × group interaction effect in any of the neuromuscular parameters. However, we found a significant large-sized time effect in all neuromuscular variables (MVC, $\eta_{p}^{2}$ = 0.181; VA_TMS_, $\eta_{p}^{2}$ = 0.250; VA_PNS_, $\eta_{p}^{2}$ = 0.250; Q_tw,pot_, $\eta_{p}^{2}$ = 0.304) as well as time to exhaustion $\eta_{p}^{2}$ = 0.601). In contrast to other studies, we could not show that a short-term SIT is able to increase endurance performance. An unchanged endurance performance after training most likely explains the lack of differences in neuromuscular variables between groups. These findings demonstrate that replication studies are needed to verify results no matter how strong they seem to be. Differences over time for the variables of neuromuscular fatigue irrespective of group (MVC, + 9.3%; VA_TMS_, + 0.2%; VA_PNS_, + 6.3%; Q_tw,pot_, + 6.3%) demonstrate test-retest effects that should be taken into consideration in future training studies and emphasize the inevitable necessity for controlled experiments.

## Introduction

High-intensity interval training (HIIT) is composed of sets of short intermittent bouts of intense physical activity interspersed with brief rest periods at low-intensity exercise (Gibala et al., 2012; Costigan et al., 2015). Sprint-interval training (SIT) is a subform of HIIT characterized by short interval durations of ∼30 s, which are performed at maximal intensity and separated by longer rest periods of 4 min (Gist et al., 2014; Weston et al., 2014; MacInnis and Gibala, 2017). Despite large differences in total training volume and training intensity compared to continuous endurance training, similar or even superior increases in aerobic capacity, determined by measuring maximal oxygen uptake [see Sloth et al. (2013), Gist et al. (2014) for review], have been documented after SIT (Burgomaster et al., 2005; Bailey et al., 2009; Hazell et al., 2010; Kavaliauskas et al., 2017; Lloyd Jones et al., 2017). Increases in aerobic performance after SIT are often linked to adaptations at the muscular level, like an enhanced muscle oxidative capacity (Barnett et al., 2004; Burgomaster et al., 2005), an increased fractional O_2_ extraction from the muscle (Bailey et al., 2009), an improved muscle microvascular structure and function (Rakobowchuk et al., 2008; Cocks et al., 2013), as well as a shift toward type IIA muscle fibers (Esbjornsson et al., 1993; De Smet et al., 2016).

However, acute bouts of intense physical activity do not only perturb homeostasis in the muscular system but also affect the central nervous system (CNS), which demonstrates detrimental effects for performance (Fernandez-del-Olmo et al., 2013; Thomas et al., 2015; Giboin et al., 2016; Hureau et al., 2016). In the present study, we use the taxonomy of fatigue as proposed by Gandevia (2001). Central fatigue corresponds to exercise-induced changes in the nervous system, which impairs the capacity to voluntarily activate the muscle. Peripheral fatigue, on the other hand, corresponds to exercise-induced changes distal to the neuromuscular junction, impairing muscle contraction (Gandevia, 2001). These short-term negative effects can most probably induce training-specific long-term neuronal adaptations (Karni et al., 1998), resulting in an improved neuromuscular performance, with or even without muscular adaptations (Moritani and deVries, 1979). We suspect that the maximal power component of SIT training could induce a similar effect. Moreover, the strength and power components of SIT could increase voluntary activation (i.e., the capacity to voluntarily activate muscles; VA), a phenomenon which is known to occur in knee extensor muscles following only four sessions of strength or power training (Giboin et al., 2018). An enhanced VA could improve the capacity of the CNS to compensate for peripheral fatigue, hence leading to an improved endurance performance.

Moreover, improvements in performance could also be explained by hypothetical training-induced changes at CNS and muscle level in an elevated tolerance of neuromuscular fatigue. This so-called “sensory tolerance limit” is a theoretical construct integrating the sum of all negative feedback from directly or indirectly utilized organs in a specific task and feedforward signals that are processed within the CNS and ultimately determine central motor drive (Hureau et al., 2018).

So far, the role of the neuromuscular system in the modification of endurance performance after HIIT or SIT have been rarely addressed. Jacobs et al. (2013) were the first to investigate if a short-term HIIT intervention could affect important components of neuromuscular fatigue. These authors demonstrated that HIIT increases aerobic capacity and aerobic performance without changes in exercise-induced peripheral fatigue, indicating an improved muscular efficiency or higher resistance to fatigue. However, the outcomes need to be interpreted cautiously as the study design lacks a control group, and results regarding the effect of exercise on voluntary activation are not reported. O’Leary et al. (2017) compared two groups following either a HIIT or a volume-matched endurance training program for 6 weeks. Both groups increased their aerobic capacity and aerobic performance during a time to exhaustion task. The HIIT group reached significantly higher exhaustion times in the endurance task than the endurance-trained group, which was accompanied by significantly attenuated levels of central fatigue but higher levels of peripheral fatigue. These results indicate that the increase in performance following HIIT could be partly induced by neural adaptations allowing a higher hypothetical “sensory tolerance limit” (Hureau et al., 2018). This result supports the assumption that neuromuscular adaptations are most probably specific to the training intensity. However, it cannot be asserted that these adaptations following HIIT also occur after SIT, where the intensity is much higher and training volume significantly lower compared to the classical HIIT protocol used by O’Leary et al. (2017). On this behalf, Lewis et al. (2017) have investigated in a non-controlled exploratory study if a cycling-based SIT intervention was capable to attenuate neuromuscular fatigue between the first and the last training session. Despite significant increases in sprint performance, no changes could be demonstrated for parameters of neuromuscular fatigue directly after the training. However, this study did perform neuromuscular measurements only directly after the sprint training sessions but not following the endurance task, which was significantly improved by the training intervention.

In the present study, we hypothesized that a SIT regimen can induce changes in CNS function that can, at least partly, explain improvements in endurance observed after such training. The training intervention was a short-term low-volume SIT protocol adopted from Burgomaster et al. (2005), known to improve endurance capacity of untrained individuals by ∼100%. This intervention was chosen because its brevity poses advantages regarding the absence of some physiological adaptations and changes in performance [i.e., long enough to induce changes at CNS level (Moritani and deVries, 1979; Giboin et al., 2018)]. To test the hypothesis that SIT has the potential to increase VA, we measured VA of the knee extensor muscles in a rested condition before and after a 2-week SIT regimen. To test the hypothesis that SIT can induce neuromuscular adaptations that lead to a higher “sensory tolerance limit,” we compared the neuromuscular function just after a fatiguing endurance task before and after the 2 weeks of SIT. If the SIT regimen can increase the “sensory tolerance limit,” we should observe a longer time to exhaustion (TTE) accompanied by a similar or even smaller decrease in VA and an accrued amount of peripheral fatigue (Zghal et al., 2015). To estimate peripheral fatigue, we can measure the reduction in muscle excitability at rest by stimulating electrically their nerve and measuring knee extensor force output (Bigland-Ritchie et al., 1986). A decrease in force output indicates a lower muscle excitability, which is a marker of peripheral fatigue. Furthermore, to better understand the mechanisms behind changes in endurance following SIT, we were interested in the location of the training-induced CNS changes. Indeed, it has been shown that training can induce changes not only at supraspinal but also at spinal level (Giboin et al., 2018; Chen et al., 2019). For this, we measured VA with TMS (VA_TMS_) and with peripheral nerve stimulation (VA_PNS_), with the former being a marker of changes at supraspinal level and the latter a marker of changes in the full nervous system (Merton, 1954; Gandevia, 2001).

## Materials and Methods

### Subjects

An *a priori* power analysis to estimate sample size was conducted using the software package G^∗^Power (Erdfelder et al., 1996). The data from the study of Burgomaster et al. (2005) were the foundation of this analysis, which indicated that a total sample size of 12 subjects is sufficient to have 95% power for detecting a large sized effect (0.596) when employing the traditional 0.05 criterion of statistical significance. Nineteen men volunteered to participate in this study. Subjects had no history of any cardiorespiratory or neuromuscular disease and were non-smokers. Training status ranged from sedentary to not specifically trained (<3 sessions/week and <1 h/session). Any form of consistent individual training routine was maintained throughout the course of the study to avoid possible effects from altered daily activity. Subjects were instructed to refrain from intense physical activity for at least 36 h and abstain from caffeine and alcohol for at least 24 h before any visit to the laboratory. This study was carried out in accordance with the recommendations of the ethical guidelines from the Ethics Committee from the University of Konstanz. The protocol was approved by the Ethics Committee from the University of Konstanz. All subjects gave written informed consent in accordance with the Declaration of Helsinki.

### Experimental Procedures

**FIGURE 1:**
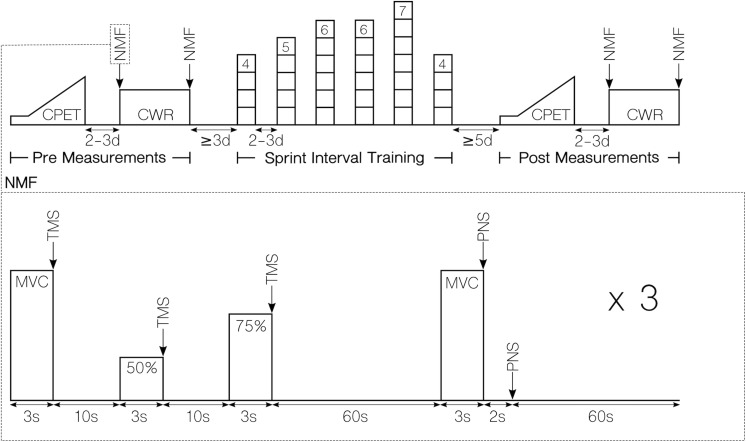
Workflow of the experimental procedures. **Top:** Overview of the complete study design for the SIT group. Except for the training intervention, procedures were identical for the CON group. **Bottom:** Procedures of for measuring neuromuscular function. CPET, cardiorespiratory exercise test; CWR, constant work rate test; NMF, neuromuscular function assessment; MVC, maximal voluntary contraction; TMS, transcranial magnetic stimulation; PNS, peripheral nerve stimulation.

The present study used a randomized matched controlled pre–post design. Subjects completed two experimental trials before and after a period of either SIT or a control period without any intervention (CON). The first experimental trial consisted of a cardiorespiratory exercise test to determine aerobic capacity. In the second experimental trial, we assessed voluntary activation and peripheral fatigue of the knee extensor muscles before and after a fatiguing constant-load protocol on a cycle ergometer. For clarity, the experimental procedures are depicted in Figure 1. In the pre-measurements, subjects were assigned to either of the two groups by an adaptive randomization procedure. Therefore, subjects were randomly allocated in batches of three to five participants to either a SIT or a CON group matched by time to exhaustion for the constant-load task. Subjects in the SIT group performed six SIT sessions in 2 weeks, while the CON group was instructed to maintain their daily activity routine for the duration of the study. Post-measurements were conducted 5 days after the last training session. The same temporal delay for pre- and post- measurements was ensured between both groups. Experimental trials as well as the SIT were all conducted under supervision. All cycling tests were performed with clipless pedals (PD-R550, Shimano, Osaka, Japan) and cycling shoes (SH-RP200, Shimano, Osaka, Japan) provided by the experimenters.

#### Cardiorespiratory Exercise Test and Familiarization

After initial anthropometrical measurements, subjects performed the cardiorespiratory exercise test on a cycle ergometer (Cyclus2, rbm elektronik-automation GmbH, Leipzig, Germany) to determine maximal oxygen uptake (VO_2__max_) with an online metabolic system (Ergostik, Geratherm Respiratory GmbH, Bad Kissingen, Germany). The cycling protocol started with a 6-min warm-up at a load of 60 W. During warm-up, the cadence could be chosen freely in the range of 80–100 revolutions per minute (rpm) but was then kept constant in the directly ensuing incremental ramp. The ramp started at a power output of 70 W and increased in steps of 5 W every 12, 15, or 20 s (15, 20, 25 W/min). Different power increments were deployed to account for variations in body weight and ensure similar exercise durations. Individual power increments were calculated by the following equation: $P\,o\,w\,e\,r\,\left(W\right)=\frac{B\,o\,d\,y\,w\,e\,i\,g\,h\,t\,\left(k\,g\right)}{4\,k\,g\times W^{-1}}$. The test was terminated when the cadence dropped below 65 rpm or the participant stopped pedaling. During the test, only cadence was displayed as augmented feedback for the subjects. In addition, two investigators provided verbal encouragement throughout the test, which was intensified after the subject reached an respiratory exchange ratio of 1. Blood lactate probes were taken (Lactate Pro II, Arkray, Kyoto, Japan) from the ear lobe directly after the termination of the protocol as well as 3 and 6 min thereafter. Respiratory gases were collected on a breath-by-breath basis. Before each test day, the gas analyzers were calibrated using ambient air and gases of known concentration (O_2_, 15.00%; CO_2_, 5.01%). Ventilatory volumes were calibrated before each test using a 3-L syringe. The metabolic cart and the ergometer were synchronized, and data were registered on a breath-by-breath basis.

To reduce variability and therefore prevent the occurrence of a systematic error in neuromuscular measurements and Wingate anaerobic test (WAnT) performance in the following experimental sessions, subjects underwent a familiarization trial ∼45 min after the cardiorespiratory exercise test. First, participants performed one WAnT after thorough instructions, followed by an orientation and familiarization procedure to the neuromuscular assessment techniques.

#### Endurance Performance Test

The second experimental session was conducted 48–72 h after the cardiorespiratory exercise test and consisted of a neuromuscular function assessment before and after a constant load cycle ergometer (SRM, Jülich, Germany) test to determine central and peripheral fatigue. Subjects warmed up by cycling for 5 min at a power output corresponding to 1.5 W/kg of body weight. After this, subjects rested for 2 min and then started the fatigue protocol, which consisted of cycling at a constant load (67% of W_max_) for as long as possible. This same relative intensity was recalculated during the post-measurements to induce identical metabolic strain on the body and elicit comparable perceptual responses compared to the pre-measurements. Based on prior experience with this methodology gathered in our laboratory, we know that this relative power output should elicit an oxygen uptake of ∼80% VO_2__peak_ and can be maintained for at least 30 min. Similar results have been shown by other investigators that demonstrated that a power output eliciting ∼80% VO_2__peak_ could be sustained for ∼26 min (Burgomaster et al., 2005). The applied load had to be sustained at a constant cadence (identical cadence than in cardiorespiratory exercise test) until subjects reached volitional exhaustion under strong verbal encouragement from three investigators. Verbal encouragement was given throughout the test and was increased when subjects reached ∼90% of their maximal heart rate. The test was terminated when subjects stopped pedaling or the cadence dropped below 65 rpm. Lactate probes were taken from the ear lobe from the beginning of the test in intervals of 5 min and directly at exhaustion. Simultaneously, we retrieved rating of perceived exertion on a 10-point scale.

#### Sprint-Interval Training

To ensure an adequate recovery, the SIT commenced at least 72 h after the last baseline measurement. The protocol for the SIT was adopted from Burgomaster et al. (2005) and consisted of six SIT sessions interspersed with 2–3 days of recovery. In each session, a series of Wingate tests were performed in an “all-out” manner. The sequence of the number of Wingate tests performed per sessions during the 2 weeks of training was 4, 5, 6, 6, 7, and 4. For every Wingate test, the SRM ergometer carried a fly-wheel mass of 9.1 kg and was set in the isokinetic mode, meaning that the ergometer kept a constant cadence independent of the subject’s power output. The preselected cadence was set at 130 rpm, as maximal power output has been reported to be achieved at a frequency around that value (Sargeant, 2007). Forty-five seconds before each Wingate test, subjects cycled at a cadence of 80 rpm and a load of 80 W. The start was introduced with a 10-s countdown after which participants were instructed to accelerate as fast as possible and continue pedaling with maximal effort throughout the 30 s. Strong verbal encouragement was given throughout the sprint. Four minutes of recovery were given after each sprint. According to individual preferences, participants were free to choose between passive or active (load, 1 W/kg) recovery in the first 3 min of the rest period. At least 1 min before the next sprint, subjects resumed pedaling. Power data from the SRM ergometer was sampled at a frequency of 2 Hz.

#### Electromyography

Before placing the electrodes, the skin was shaved, lightly abraded, and cleaned with alcohol swabs. Wireless surface EMG electrodes (Trigno Lab, Delsys Inc., Natick, MA, United States) with an interelectrode distance of 10 mm and an electrode size of 1 × 5 mm were then placed on the muscle bellies of the vastus lateralis and the biceps femoris of the right leg. The position and orientation of the electrodes were consistent with the SENIAM guidelines (Hermens et al., 1999). EMG signals were bandpass filtered between 20 and 450 Hz, amplified by 1 kV/V and sampled at a rate of 4 kHz and registered with an analog-to-digital board (Micro 1401, Cambridge Electronic Design Limited, Cambridge, United Kingdom) on a desktop PC.

#### Muscle Force

Muscle force was measured during isometric knee extension in a seated position with the hip and knee flexed in a 90° angle. Two centimeters above the lateral malleolus, the right leg of the subject was connected to a force transducer (9321A, Kistler, Winterthur, Switzerland) by a cuff and a non-compliant tension belt. The cuff position was marked with indelible ink for an exact relocation during the experiment. To restrict body displacement during contractions, the subject was further attached to the custom-built chair with tension belts at the hip (with paddings) and round the chest. Subjects folded their arms in front of their chest during each contraction to limit force enhancement by holding onto the chair. Before beginning the neuromuscular function assessment, participants performed a standardized warm-up protocol consisting of 13 isometric contractions at increasing intensity. Two minutes after the warm-up, two MVCs were performed with a recovery time of 1 min between each contraction. If the force between the two contractions varied more than 5%, a third MVC was conducted after 2 min of rest. The highest MVC was registered and taken as the highest isometric force the subject could attain. In all MVC measurements thereafter, the target force was set ∼2% above the achieved MVC. In addition, the investigators gave strong verbal encouragement during the MVC. MVCs that were conducted before the fatiguing cycle protocol and were not regarded as maximal by the subject or the main investigator were discarded and repeated. Force data were sampled and registered simultaneously and with the identical settings than the EMG recordings.

#### Peripheral Nerve Stimulation

Single rectangular electrical pulses (1 ms) were delivered via custom-built surface electrodes to the femoral nerve of the right leg by a constant-current stimulator (DS7A, Digitimer, Hertfordshire, United Kingdom) to elicit maximal M waves (M_max_) in VL. The cathode (5 cm^2^) was carefully placed on the femoral nerve in the femoral triangle, and the anode (24 cm^2^) was positioned on the center of the m. gluteus maximus. The optimal electrode position was identified when a submaximal stimulus elicited a clear and pronounced biphasic M-wave signal. The position was marked with indelible ink to ensure identical electrode placement for pre- and post-measurements. The stimulation intensity to evoke M_max_ was identified by searching the lowest current output that would elicit the highest M-wave amplitude. To ensure supramaximal stimulus intensity during VA measurements, the current output of the stimulator was increased to 150% of the lowest current output able to elicit M_max_.

#### Transcranial Magnetic Stimulation

Single biphasic pulses were delivered by a concave figure-8 coil (97 mm outside diameter) over the left motor cortex to evoke MEPs in the right VL (Magpro R30, MagVenture, Farum, Denmark). The handle of the coil was oriented perpendicular to the interhemispheric fissure, with its center positioned over the left hemisphere a few centimeters lateral from the vertex. While holding a contraction of 5% of MVC and setting maximal stimulator output to 60%, the coil position was adjusted to elicit the largest possible MEP in VL while concomitantly eliciting a MEP in biceps femoris smaller than 10% of the MEP in VL. The position was marked with indelible ink on the scalp to ensure the identical localization of the MEP hotspot throughout the experiment. During VA_TMS_ measurements, stimulus intensity was set to elicit the largest possible MEP (90–100% maximal stimulator output) and was kept constant throughout the protocol.

#### Neuromuscular Function

To determine acute fatigue- and training-induced adaptations of the CNS, we measured voluntary activation of the neuromuscular system by the twitch interpolation technique (Merton, 1954). Here, the additional force (superimposed twitch) evoked by a single PNS at maximal force during an MVC is expressed as a fraction of the force produced by an identical stimulus at rest of the potentiated muscle ∼2 s after the contraction (potentiated resting twitch). The equation: $VA_{\text{PNS}}\left(\%\right)=\left(1-\frac{s\,u\,p\,e\,r\,i\,m\,p\,o\,s\,e\,d\,t\,w\,i\,t\,c\,h}{p\,o\,t\,e\,n\,t\,i\,a\,t\,e\,d\,r\,e\,s\,t\,i\,n\,g\,t\,w\,i\,t\,c\,h}\right)\times 100$ expresses the relative level of voluntary drive. A reduction in VA_PNS_ demonstrates the presence of fatigue due to processes proximal to the neuromuscular junction, often referred to as central fatigue (Gandevia, 2001). Peripheral fatigue indicates fatigue due to processes occurring distal from the neuromuscular junction and is demonstrated by reductions in twitch force from PNS of the potentiated muscle at rest.

For further specification of the site of fatigue within the CNS, TMS of the motor cortex is used to measure VA by the twitch interpolation technique (Todd et al., 2003) and validated for knee extensor muscles by Goodall et al. (2009) and Sidhu et al. (2009). Briefly, resting twitch amplitude is estimated by linear extrapolation from the amplitude of three superimposed twitches evoked by TMS at different contraction intensities (50, 75, 100%) of the target muscle. Together with the single TMS during maximal contraction, we quantified the extent of motor cortical drive by the following equation:${\mathrm{VA}}_{\text{TMS}}\left(\%\right)=\left(1-\frac{\mathrm{superimposed}\,\mathrm{twitch}}{\mathrm{estimated}\,\mathrm{resting}\,\mathrm{twitch}}\right)\times 100$. A reduction in VA_TMS_ is called supraspinal fatigue since impairments occur “at or above the level of motor cortical output” (Sidhu et al., 2009) and is considered a subset of central fatigue (Gandevia, 2001).

The experimental procedure (see Figure 1) started 3 min after the warm-up protocol. At first, we measured VA_TMS_ for which subjects performed three isometric leg extensions at 100, 50, and 75% of MVC, each contraction lasting ∼3 s with a rest period of 10 s. At each of the contractions, a TMS was given at identical stimulator output level. After 1 min of recovery, VA_PNS_ was measured. For this, PNS was delivered to the motor nerve during an MVC and 2 s after the contraction. VA_TMS_ and VA_PNS_ measurement procedures were repeated three times each before and after the endurance task. At each contraction, feedback of performance was administered by visual representation of the force curve on a computer monitor. For motivational reasons, strong verbal encouragement from two investigators was given to the subject during each MVC. If subjects or the investigators identified an MVC as submaximal, they were able to discard and repeat the MVC after one more minute of recovery. This situation occurred three times for two subjects during the complete data collection period.

### Data Analysis

Respiratory gases and power data from the cardiorespiratory exercise test were sampled on a breath-by-breath basis, then interpolated to 1-s intervals and filtered in MATLAB (R2016b, Mathworks, Natick, MA, United States) with a standard Gaussian filter [kernel: ${\left(\sigma \,\sqrt{2\,n}\right)}^{-1}\,e\,x\,p\,\left(-0.5\,t/\sigma^{2}\right)$ with σ = 20 s for respiratory and σ = 3 s for power data). The highest VO_2_ value and the highest power output obtained after the smoothing procedure were defined as VO_2__max_ and maximum power (Power_max_), respectively.

The power data assessed during the Wingate test from the SRM ergometer was averaged to 1-s intervals and analyzed for peak power, mean power, and fatigue index. Peak power was defined as the highest power output over one of the 1-s intervals. Mean power was calculated as the average power from time of peak power until the end of the test. The fatigue index was defined as the decrement in power output calculated from peak power until the end of the test. Average peak power, mean power, and fatigue index were calculated from the four Wingate tests for the first and the sixth SIT training session.

The mean blood lactate level in the endurance task was calculated for each participant. All lactate values starting from 10 min into the endurance test until the last value with a full 5-min interval were taken into account for the analysis. Neuromuscular function variables were MVC, VA_TMS_, and VA_PNS_, and Q_tw,pot_.

### Statistics

Statistical analyses were performed with JASP (Version 0.8.6) and R (Version 3.3.3) in RStudio (Version 1.0.143). A three-way repeated measures ANOVA was performed for all neuromuscular measurements to identify possible interaction effects between three independent variables, being *time training* (within-subject factor) referring to the time before and after the training period, *time task* (within-subject factor) referring to the time before and after the exercise bout, and *group* (between-subject factor) referring to the treatment groups.

A two-way repeated measures ANOVA was performed for all exercise performance variables and the anthropometric variables weight and BMI to identify possible interaction effects between time training (within-subject factor) and group (between-subject factor).

Within-subject data with more than two levels was tested for the assumption of sphericity with Mauchly’s test of sphericity. If sphericity was violated, we applied the Greenhouse – Geisser correction. Between-subject data were tested for the assumption of homogeneity of variances with the Levene’s test. No data set showed significant results in the Levene’s test. Main effects and interactions were analyzed *post hoc* using paired *t*-tests with Bonferroni–Holm correction. Anthropometric variables height and age were analyzed for differences with independent samples *t*-tests. The level of significance was set to *p* < 0.05. Data are presented as means ± SD. Effect sizes are reported as $\eta_{p}^{2}$. Effect sizes are defined according to Cohen (1988), a small effect size being $\eta_{p}^{2}$ 0.01, a medium effect being 0.01–0.06, and a large effect being >0.14.

## Results

In total, 31 subjects were recruited for this study. Ten subjects dropped out before finishing the pre-measurements due to lack of motivation (*n* = 5), vast discomfort from the stimulation procedures (*n* = 3), or knee pain during the isometric contractions (*n* = 2). Two subjects of the SIT group dropped out during the training intervention due to illness. In total, 19 subjects were considered for data analysis (*n* = 9 SIT, *n* = 10 CON). The SIT group completed 288 from 288 scheduled Wingate tests.

### Anthropometry

The *t*-tests for height and age as well as the ANOVA for weight and BMI showed no significant difference between the training and control group. Anthropometric measurements are presented in Table 1.

**TABLE 1:** Results for the endurance task and the cardiorespiratory exercise test (CPET) for pre- and post- training in SIT and CON.

	SIT		CON		*t*-test		
Anthropometry	Pre	Post	Pre	Post			
Height (cm)	176 ± 7	−	177 ± 8	−	0.842		
Age (years)	25.1 ± 3.4	−	26.3 ± 5.5	−	0.586		

					**RM ANOVA**		
					**Time**	**Group**	**Inter.**

Weight (kg)	78.8 ± 10.3	78.5 ± 10.2	73.8 ± 11.4	74.5 ± 11.9	0.566	0.390	0.137
BMI (kg/m^2^)	25.5 ± 3.6	25.4 ± 3.7	23.6 ± 2.6	23.8 ± 2.7	0.548	0.233	0.177
**Endurance task**							
TTE (min:s)	43:00 ± 16	50:38 ± 25	46:49 ± 24	45:26 ± 25	0.324	0.945	0.159
Power_mean_ (W)	204 ± 24	210 ± 24	190 ± 19	190 ± 19	0.166	0.093	0.172
Work (kJ)	532 ± 205	622 ± 283	533 ± 293	517 ± 308	0.319	0.678	0.161
Heart rate_mean_ (bpm)	174 ± 11.2	166 ± 9.4	172 ± 7.9	167 ± 8.0	**<0.001**	0.832	0.156
Heart rate_max_ (bpm)	188 ± 9.0	179 ± 10.4	186 ± 8.2	179 ± 8.8	**<0.001**	0.750	0.555
Lactate_mean_ (mmol/L)	7.7 ± 2.1	6.1 ± 2.5	6.9 ± 2.7	5.4 ± 1.9	**<0.001**	0.469	0.977
Lactate_exh_ (mmol/L)	10.1 ± 3.7	7.1 ± 3.7	8.5 ± 4.1	6.4 ± 2.8	**<0.001**	0.499	0.400
**Cardiorespiratory exercise test**							
TTE (min:s)	12:08 ± 2	12:21 ± 1	12:14 ± 2	12:15 ± 2	0.343	0.992	0.421
Power_max_ (W)	306 ± 34	312 ± 37	284 ± 27	284 ± 28	0.252	0.091	0.252
Power_max_ (W/kg)	3.9 ± 0.56	4.0 ± 0.45	3.9 ± 0.47	3.9 ± 0.45	0.508	0.672	0.115
VO_2__max_ (ml/min/kg)	45.2 ± 6.6	45.3 ± 5.0	44.4 ± 5.0	43.1 ± 4.7	0.321	0.548	0.195
RER_max_	1.32 ± 0.04	1.29 ± 0.06	1.31 ± 0.08	1.33 ± 0.07	0.460	0.652	0.068
Heart rate_max_ (bpm)	194 ± 9	189 ± 10	193 ± 6	190 ± 8	**<0.001**	1.000	0.659
Lactate_exh_ (mmol/L)	11.9 ± 2.5	10.6 ± 2.4	9.2 ± 4.1	9.4 ± 2.5	0.273	0.171	0.153
Lactate_exh+__3__^´_ (mmol/L)	14.5 ± 1.3	12.6 ± 2.1	12.2 ± 2.5	11.2 ± 2.4	**0.005**	0.060	0.347
Lactate_exh+__6__^´_ (mmol/L)	14.3 ± 2.2	10.9 ± 3.4	11.0 ± 2.4	9.5 ± 2.1	**<0.001**	**0.039**	0.126

*Values are mean ±SD. TTE, time to exhaustion; VO_2max_, maximal oxygen uptake; RER_*max*_, respiratory exchange ratio; exh, time at exhaustion; inter., interaction effect; statistics are *p*-values. The bold values are demonstrating a significant result.*

### Sprint-Interval Training

Maximal power (860 ± 185 W vs. 928 ± 183 W) and mean power (453 ± 89 W vs. 493 ± 70 W) of the Wingate tests improved significantly from the first to the sixth training session in the SIT group (*p* = 0.002 and *p* = 0.039, respectively). No significant effect was found for Fatigue Index and maximal heart rate.

### Cardiorespiratory Exercise Test

The ANOVA demonstrated no significant interaction or group effect for any variable assessed for the cardiorespiratory exercise test. Main effects for time were found in maximal heart rate, which was significantly reduced in the post-measurements (see Table 1). Blood lactate values 3 and 6 min into recovery were significantly lower in the post-measurements (see Table 1). Blood lactate values at exhaustion were not significantly altered. Cardiorespiratory exercise test data are presented in Table 1.

### Endurance Performance Test

**FIGURE 2:**
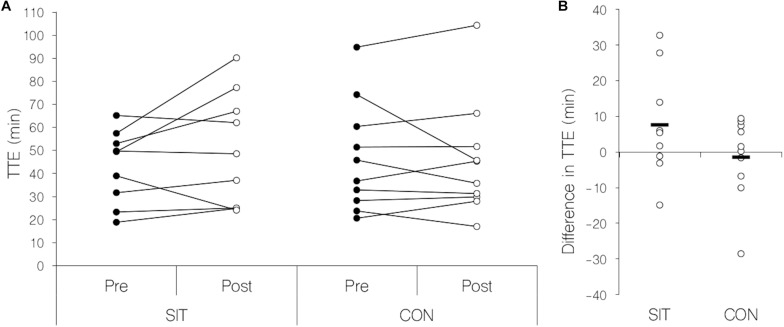
Scatterplots for individual measurements of time to exhaustion in the endurance task. **(A)** Change in time to exhaustion before and after the intervention separated by training group. Filled circles: pre-training condition, open circles post-training condition. **(B)** Difference in time to exhaustion between pre- and post- measurements separated by training group. Black line: Mean difference for each group; open circles: individual change in time to exhaustion. SIT, sprint-interval training group; CON, control group; Pre, before training intervention; Post, after training intervention; TTE, time to exhaustion.

There was no significant interaction or main effect for group in time to exhaustion or any other parameter obtained in the endurance performance test (see Figure 2). Main effects for time were found in maximal and mean heart rates (see Table 1), these parameters being significantly reduced in the post-measurements. Mean blood lactate values and blood lactate at exhaustion were significantly elevated in the post-measurements demonstrated by a significant main effect for time (see Table 1). Results for the endurance performance task are presented in Table 1.

### Neuromuscular Function

We measured neuromuscular function before as well as on several time points after the endurance task. For clarity, we refer to this as time task, whereas time training refers to the time either before or after the training/control period. Results of the neuromuscular parameters are presented in Table 2.

**TABLE 2:** Mean and SDs for variables of neuromuscular function parameters measured before and after the endurance task for pre- and post- training.

	SIT		CON	
MVC (*N*)*	Pre	Post	Pre	Post
Pre end. task	542 ± 100	555 ± 101	550 ± 114	574 ± 106
Post end. task 2′	438 ± 93	453 ± 103	463 ± 109	490 ± 92
Post end. task 3′	410 ± 96	465 ± 101	432 ± 73	460 ± 82
Post end. task 4′	409 ± 85	464 ± 95	421 ± 94	464 ± 85
Post end. task 5′	414 ± 84	474 ± 98	434 ± 77	481 ± 110
Post end. task 6′	417 ± 92	481 ± 102	433 ± 99	487 ± 97
Post end. task 7′	424 ± 95	474 ± 108	445 ± 84	492 ± 100
VA_TMS_ (%)*				
Pre end. task	97.3 ± 2.4	96.1 ± 2.8	98.7 ± 1.7	97.9 ± 2.0
Post end. task 2′	86.3 ± 13.3	80.5 ± 19.2	95.2 ± 5.1	92.4 ± 9.4
Post end. task 4′	87.4 ± 10.5	88.5 ± 6.5	86.9 ± 7.3	94.1 ± 4.1
Post end. task 6′	88.6 ± 7.8	91.9 ± 3.1	93.8 ± 5.2	95.7 ± 3.8
VA_PNS_ (%)*				
Pre end. task	90.9 ± 4.5	92.2 ± 3.3	94.0 ± 4.3	94.4 ± 3.6
Post end. task 3′	81.8 ± 8.7	88.3 ± 6.2	86.3 ± 3.8	90.0 ± 3.4
Post end. task 5′	83.5 ± 6.4	88.4 ± 6.0	87.1 ± 5.3	90.7 ± 3.9
Post end. task 7′	83.6 ± 6.5	86.4 ± 8.9	86.9 ± 6.0	89.0 ± 5.2
Q_tw,pot_ (*N*)*				
Pre end. task	137 ± 27	131 ± 22	124 ± 37	128 ± 33
Post end. task 3′	82 ± 24	91 ± 27	82 ± 24	88 ± 19
Post end. task 5′	84 ± 23	92 ± 24	81 ± 24	88 ± 22
Post end. task 7′	84 ± 23	94 ± 23	79 ± 25	88 ± 23

Values are mean ±SD *A main effect for time (*p* < 0.05), end, endurance. For VA_*TMS*_, *n* = 14 (*n* = 6 SIT; *n* = 8 CON); for VA_*PNS*_ and Q_*tw,pot*_, *n* = 17 (*n* = 8 SIT; *n* = 9 CON).

There was no interaction effect or main effect for group in any of the neuromuscular parameters. However, we observed significant interaction effects in time task × time training in all neuromuscular variables tested [MVC, *F*(2.6,43.8) = 3.747, *p* = 0.022, $\eta_{p}^{2}$ = 0.181; VA_TMS_, *F*(1.82,21.84) = 4.007, *p* = 0.036, $\eta_{p}^{2}$ = 0.250; VA_PNS_, *F*(1.82,21.84) = 4.007, *p* = 0.036, $\eta_{p}^{2}$ = 0.250; Q_tw,pot_, *F*(1.80,28.74) = 8.119, *p* = 0.002, $\eta_{p}^{2}$ = 0.304]. *Post hoc* analysis revealed that, for MVC, these interactions were explained by significantly higher values in all measurements during the recovery from the endurance task, demonstrating less fatigue in the post-test in both groups. Similar results were found for Q_tw,pot_ at 5 and 7 min into recovery, indicative of better fatigue recovery in both groups. No significant differences in *post hoc* comparisons were found for VA_TMS_ and VA_PNS_.

## Discussion

The present study investigated the effects of a short-term low-volume SIT on neuromuscular function of the knee extensors in an endurance task. Six sessions of SIT were not sufficient to significantly increase endurance performance, making the between group comparison of the neuromuscular variables obsolete. However, we found for both groups clear differences in neuromuscular variables after the intervention indicative of a test – retest effect.

It was unexpected to find no increase in endurance performance for the training group since we adopted a similar training and testing protocol that reported marked improvements in endurance performance (Burgomaster et al., 2005). These diverging outcomes might be a result of some methodological variations. First, Burgomaster et al. (2005) seemingly failed to allocate subjects to groups by a randomization procedure, increasing the chance of systematic bias. In this study, subjects were randomly assigned to either of the two groups by an adaptive randomization procedure. Second, the groups of the present study consisted of the male sex only, whereas the study of Burgomaster et al. (2005) included persons of both sexes. We have not included women due to the intimate nature of the electrical stimulation protocol (placing of the electrodes in the femoral triangle). However, there is ample evidence that women and men have similar adaptive profiles after endurance and HIIT training (Carter et al., 2001; Astorino et al., 2011). In addition, to the best of our knowledge, all short-term (≤2 weeks) SIT studies implementing an endurance performance task have consistently reported significant improvements (Burgomaster et al., 2005, 2006; Gibala et al., 2006; Babraj et al., 2009; Hazell et al., 2010; Jacobs et al., 2013; Lewis et al., 2017). Furthermore, independent of the training intervention duration and specificity of the protocol, a meta-analysis reported consistent results for improvements in endurance performance after a SIT (Sloth et al., 2013).

The most relevant observation from our neuromuscular measurements was that the control group improved consistently in all neuromuscular variables, demonstrating a considerable test – retest effect. This finding indicates that the two exhausting cycling tests during the pre-measurements might be sufficient to induce neuromuscular adaptations – at least on a short time scale of ∼2 weeks. Therefore, future training studies implementing similar methods to measure neuromuscular function should consider that the variables of interest might underlie a test – retest effect. We emphasize the need for a control group in these types of studies to allow for a reliable interpretation of possible neuromuscular adaptations following any intervention.

The significant amount of neuromuscular fatigue induced by the endurance task which we found in our data (see Table 2) is in line with other studies. O’Leary et al. (2017), Sidhu et al. (2017), and Aboodarda et al. (2018) demonstrated a relative reduction in VA_TMS_ and VA_PNS_ by ∼3–8% and ∼3–14% after an exhaustive endurance task, which is quite similar to a 4–11% and 4–10% reduction of VA_TMS_ and VA_PNS_ observed in the present study. The reductions in MVC (15–20%) observed in the present study is in line with previous works that showed reductions of ∼11–23% (O’Leary et al., 2017; Sidhu et al., 2017). The reductions in peripheral muscle fatigue in the present study (31–40%) were comparable to other studies, demonstrating reductions of ∼30–40% (O’Leary et al., 2017; Sidhu et al., 2017). Recovery kinetics from neuromuscular fatigue after endurance exercise training or after single bouts of cycling exercise have been investigated very scarcely. Sidhu et al. (2017) have shown that MVC, peripheral fatigue, and VA_PNS_ remain significantly reduced for at least 4 min after exercise cessation. Zghal et al. (2015) have shown that VA_TMS_ and VA_PNS_ started to recover (or had already been recovered) 3–9 min after a low-force isometric contraction to exhaustion. We show that VA_PNS_ and VA_TMS_ remain significantly reduced for up to 7 and 6 min, respectively. In line with the study by Zghal et al. (2015) who showed reduced MVC and twitch responses for more than 33 min after a fatiguing task, we were able to follow similar reductions up to 7 min. Although our results are in line with other studies, direct comparisons should be made with great care since fatigue protocols and task modalities vary substantially between the above-mentioned studies.

The significantly lower maximal heart rate values in both post-tests in the present study could suggest that subjects did not reach their maximal tolerance limit due to motivational reasons. However, unaltered maximal lactate values in the cardiorespiratory exercise test and higher lactate values in the endurance task indicated that this was not the case. In addition, we demonstrate that maximal rating of perceived exertion at exhaustion in the endurance task and respiratory exchange ratio at exhaustion in the ramp test were similar between pre- and post- measurements, indicating that subjects reached their maximal effort during these trials. Lower maximal heart rate after a training regime is a known phenomenon [see Zavorsky (2000) for review] that can be explained by augmented stroke volume as a result of acute increases in blood volume (Convertino, 1983) and enhanced baroreceptor reflex function (Somers et al., 1991; Kingwell et al., 1992).

## Limitations

Two methodological differences between this study and the study from Burgomaster et al. (2005) represent limitations regarding the replication of results. First, different methods for determining exercise intensity in the endurance task were used. While we opted to set a fixed power output relative to the peak power achieved in the ramp test, Burgomaster et al. (2005) have set an intensity that corresponds to ∼80% of VO_2__max_. However, in unpublished data from our laboratory, we observed that, in average, 67% of the maximum power output during a ramp test that lasts 12–15 min evoked an oxygen uptake during a constant workload exercise that corresponds to ∼79% of VO_2__max_. Considering that aerobic capacity between the cohorts of the two studies was similar, we could assume relative intensity to be comparable as well. However, when comparing TTE of the pre-measurements between the two studies, it becomes apparent that relative intensity in our study must have been lower since TTE was nearly twice as high in our study. This implies that reliance on aerobic energy pathways must have been more pronounced in our study than in the study of Burgomaster et al. (2005). To the best of our knowledge, there is no other SIT study implementing an endurance task of such long duration. We therefore assume that SIT might not show an effect in endurance tasks that rely pre-dominantly on aerobic energy metabolism despite beneficial physiological adaptations for these kinds of exercises demonstrated in previous studies (Barnett et al., 2004; Burgomaster et al., 2005; Rakobowchuk et al., 2008; Bailey et al., 2009; Cocks et al., 2013). A second methodological difference between the two studies that could have an effect on time to exhaustion in the endurance task was that an adjustment of exercise intensity in the post-measurements was only applied in this study. Here, we normalized the intensity of the endurance task to account for the increase in aerobic fitness, whereas Burgomaster et al. (2005) used the identical power output for the pre- and post- measurements. The rationale behind this was that we aimed to induce an identical metabolic strain and perceptual responses in the post-measurements compared to the pre-measurements, which would not have been the case if we would have chosen the same absolute power output. Although we made only small adjustments to power output (+6 W in SIT, 0 W in CON), the question is, if they can account for the large discrepancies in TTE improvement (+18% in the present study vs. +100% in the study of Burgomaster et al. (2005). To the best of our knowledge, only one study compared TTE improvements for an endurance task between normalized and same absolute intensity after a training regime (O’Leary et al., 2017). Compared to pre-measurements, TTE in the endurance task accomplished at 78–80% VO_2__max_, was significantly improved by 43% with normalized intensity and even 148% using the same absolute intensity. Therefore, it might be possible that this methodological variation can account for large differences in TTE in comparable groups of subjects.

## Conclusion

Two weeks of SIT were not sufficient to induce increases in endurance performance as shown in other studies. These findings demonstrate that very modest variations in methodology can lead to severely contrasting outcomes and that replication studies are needed to verify results no matter how strong they seem to be. We share the opinion of Halperin et al. (2018) that sport and exercise science in general needs to address this problem.

Time effects in all neuromuscular parameters and in heart rate and blood lactate values demonstrate strong test – retest effects that should be taken into consideration in future (short-term) training studies and emphasize the absolute need for controlled experiments. It is possible that the effect of the training was confounded with the effect of the two pre-training measurements. We speculate that in this situation, a longer intervention would have been necessary to elicit adaptations discernible from the adaptations induced by the pre-measurements.

## Data Availability Statement

The raw data supporting the conclusions of this article will be made available by the authors, without undue reservation, to any qualified researcher.

## Ethics Statement

This study was carried out in accordance with the latest revision of the declaration of Helsinki and specifically approved by the Ethics Committee under no. 06/2019 of the University of Konstanz. All subjects gave written informed consent before participating in the study.

## Author Contributions

RB conceived, planned, carried out the experiments, performed the data analysis, and wrote the first draft of the manuscript. L-SG and MG contributed to the conceptual refinement of the study and supervised the findings of this work. All authors discussed the results and contributed to the final manuscript and manuscript revision.

## Conflict of Interest

The authors declare that the research was conducted in the absence of any commercial or financial relationships that could be construed as a potential conflict of interest.
